# Motor-related brain activity during action observation: a neural substrate for electrocorticographic brain-computer interfaces after spinal cord injury

**DOI:** 10.3389/fnint.2014.00017

**Published:** 2014-02-19

**Authors:** Jennifer L. Collinger, Ramana Vinjamuri, Alan D. Degenhart, Douglas J. Weber, Gustavo P. Sudre, Michael L. Boninger, Elizabeth C. Tyler-Kabara, Wei Wang

**Affiliations:** ^1^Human Engineering Research Laboratories, Department of Veterans AffairsPittsburgh, PA, USA; ^2^Department of Physical Medicine and Rehabilitation, University of PittsburghPittsburgh, PA, USA; ^3^Department of Bioengineering, University of PittsburghPittsburgh, PA, USA; ^4^Program in Neural Computation, Carnegie Mellon UniversityPittsburgh, PA, USA; ^5^Clinical and Translational Science Institute, University of PittsburghPittsburgh, PA, USA; ^6^Department of Neurological Surgery, University of PittsburghPittsburgh, PA, USA

**Keywords:** BCI, motor cortex, action execution, action observation, electrocorticography (ECoG), mirror neurons, spinal cord injury

## Abstract

After spinal cord injury (SCI), motor commands from the brain are unable to reach peripheral nerves and muscles below the level of the lesion. Action observation (AO), in which a person observes someone else performing an action, has been used to augment traditional rehabilitation paradigms. Similarly, AO can be used to derive the relationship between brain activity and movement kinematics for a motor-based brain-computer interface (BCI) even when the user cannot generate overt movements. BCIs use brain signals to control external devices to replace functions that have been lost due to SCI or other motor impairment. Previous studies have reported congruent motor cortical activity during observed and overt movements using magnetoencephalography (MEG) and functional magnetic resonance imaging (fMRI). Recent single-unit studies using intracortical microelectrodes also demonstrated that a large number of motor cortical neurons had similar firing rate patterns between overt and observed movements. Given the increasing interest in electrocorticography (ECoG)-based BCIs, our goal was to identify whether action observation-related cortical activity could be recorded using ECoG during grasping tasks. Specifically, we aimed to identify congruent neural activity during observed and executed movements in both the sensorimotor rhythm (10–40 Hz) and the high-gamma band (65–115 Hz) which contains significant movement-related information. We observed significant motor-related high-gamma band activity during AO in both able-bodied individuals and one participant with a complete C4 SCI. Furthermore, in able-bodied participants, both the low and high frequency bands demonstrated congruent activity between action execution and observation. Our results suggest that AO could be an effective and critical procedure for deriving the mapping from ECoG signals to intended movement for an ECoG-based BCI system for individuals with paralysis.

## Introduction

Brain computer-interfaces (BCIs) transform brain activity into control signals for external devices including computers, communication aids, and prostheses (Wolpaw et al., 2002). Recent studies have demonstrated that BCIs may be able to restore functions that cannot be completed with the paralyzed upper limbs (Hochberg et al., 2012; Collinger et al., 2013b). The key component of a motor-BCI system is its neural decoder, or neuro-motor map, which is a set of decoding weights that transform motor cortical activity to intended movement. Typically, decoding weights can be calculated using motor cortical activity and the associated overt movement (Taylor et al., 2002; Wang et al., 2010a). However, this is not practical for clinical BCI systems as the targeted BCI users are often individuals who are unable to generate overt movement due to conditions such as spinal cord injury (SCI), amyotrophic lateral sclerosis (ALS), or upper limb amputation. An alternative strategy for identifying the neuro-motor map is to use motor cortical activity associated with action observation (AO) (Tkach et al., 2008; Velliste et al., 2008; Dushanova and Donoghue, 2010). Previous studies using magnetoencephalography (MEG) and electroencephalography (EEG) have shown a decrease in sensorimotor rhythm (10–30 Hz) power during both action execution and observation (Hari et al., 1998; Muthukumaraswamy et al., 2004; Caetano et al., 2007; Perry and Bentin, 2009; Press et al., 2011). Similarly, many motor cortical neurons recorded with microelectrodes show comparable firing rates during both conditions (Tkach et al., 2007; Dushanova and Donoghue, 2010). This suggests that AO may be used to identify the neuro-motor map without the need for overt movement.

Electrocorticography (ECoG) is a promising neural recording modality for BCI applications (Leuthardt et al., 2004; Schalk et al., 2007; Acharya et al., 2010; Miller et al., 2010; Flint et al., 2013; Wang et al., 2013). ECoG-based BCIs typically generate movement commands based on changes in ECoG signal power in specific frequency bands, including the sensorimotor rhythms and high-gamma band (>60 Hz). The sensorimotor rhythms typically show decreased spectral power during overt movement, and increased spectral power is observed in the high-gamma band (Crone et al., 1998a,b; Miller et al., 2007). It has been suggested that the high-gamma band carries rich movement information for BCI control, such as hand movement direction (Leuthardt et al., 2004; Heldman et al., 2006) and individual finger movement (Kubánek et al., 2009; Miller et al., 2009; Wang et al., 2009). However, there has not been any report to date that characterized high-gamma band modulation by observed hand movement using cortical surface recording in humans. Therefore, this study aims to determine whether action observation-related activity can be measured using ECoG during observed hand movement in both able-bodied subjects and an individual with tetraplegia due to SCI. We expect that significant and congruent motor-related cortical activity, defined as decreased spectral power of the sensorimotor rhythm and increased spectral power of the high-gamma band, will be recorded during execution and observation of grasping movements.

## Materials and methods

### Participants

Four able-bodied subjects (F, G, I, and J) were recruited to participate in this study. All subjects were undergoing subdural ECoG monitoring for intractable epilepsy and were able to perform simple behavioral tasks and follow instructions as determined by the investigators. None of these participants had any motor impairment. We also collected data from an individual with cervical SCI (Subject S) who had an ECoG array implanted over his sensorimotor cortex for a BCI study (Wang et al., 2013). This trial is registered at clinicaltrials.gov (NCT01393444). Informed consent was provided by all subjects prior to participation in this research, which was approved by the Institutional Review Board at the University of Pittsburgh.

**Figure 1:**
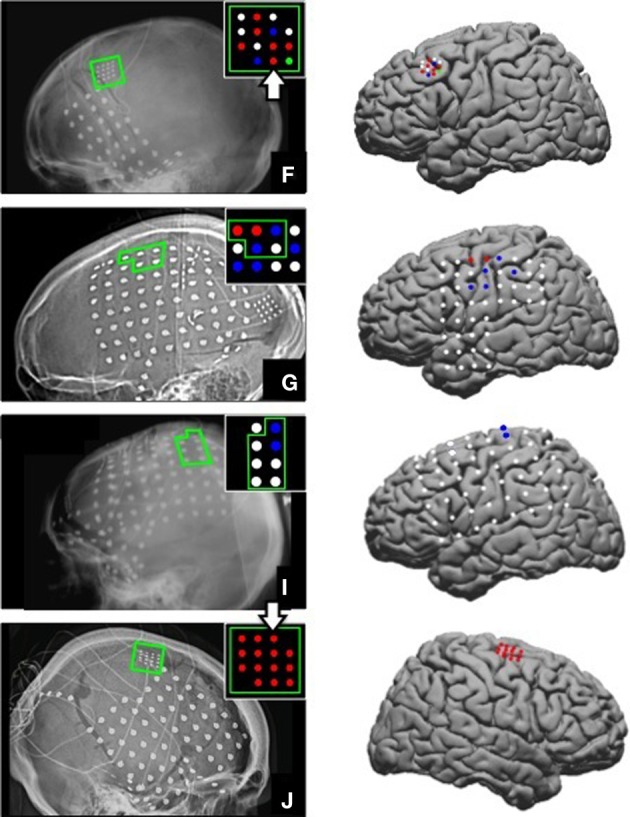
**ECoG grid locations**. Left, ECoG grid locations shown on x-ray for Subjects F, G, I, and J. The motor cortical areas are highlighted in green. The schematic inset on each x-ray shows electrodes that were significantly modulated as indicated by the color. Red = Significant modulation during action execution (AE) and observation (AO), Blue = Significant modulation during AE only, Green = Significant modulation during AO only, White = no significant modulation. Electrode positions are shown on a standard brain model to the right of each x-ray using the same color scheme. The two large white arrows (Subjects F and J) mark the two electrodes whose data are shown in Figures 3, 4.

The ages of the able-bodied participants (F, G, I, and J) were 17, 23, 12, and 45 respectively. They all had a standard ECoG grid (Ad-Tech Medical Instrument Corporation, Racine, WI), with 3 mm diameter platinum disc electrodes and 10 mm center-to-center spacing, implanted to localize seizure foci prior to resection surgery. In addition, 2 subjects (F and J) were implanted with a small high-density ECoG grid (Ad-Tech Corp., Racine, WI) placed adjacent to the clinical grid as part of the research protocol with the goal of targeting motor cortical areas. The high-density ECoG grid contained 16 platinum disc electrodes with 1.5 mm diameter and 4 mm center-to-center spacing. Two corner electrodes on the high-density ECoG grid served as reference channels. Grid locations for the 4 able-bodied subjects are shown on x-ray and plotted on a standard brain model in Figure 1. The Location on Cortex (LOC) toolbox was used to compute the electrode position in Talaraich coordinates based on the lateral x-ray and to render these positions on a standard brain model (Miller et al., 2007). The current study focused on data collected from the high-density ECoG electrodes targeted to motor cortical areas for Subject F and J and data collected from standard ECoG electrodes over the motor cortical areas for Subject G and I. The high-density ECoG grids were targeted to motor cortical areas based on anatomical landmarks visible on the exposed cortical surface during the implantation surgery. Cortical stimulation mapping through the standard ECoG grids was performed as part of the clinical procedure to identify motor cortical areas highlighted in Figure 1 for Subjects G and I. No cortical stimulation was performed for the high-density research grid as this was not part of the standard clinical procedure.

Subject S was a 30-year-old right-handed male with a complete C4 level SCI that occurred 7 years prior to participating in this study. The participant had no volitional arm or hand movement. A custom high-density ECoG grid (PMT Corp, Chanhassen, MN, USA) was implanted over the left sensorimotor cortex using stereotactic image guidance as shown in Wang et al. (2013). The grid was placed over cortical areas activated by video-guided attempted movement as measured with functional magnetic resonance imaging (fMRI) and MEG. The high-density ECoG grid contained 32 platinum disc electrodes with 28 electrodes facing the brain for recording and 4 ground and reference electrodes facing the dura. Two recording electrodes and 2 ground electrodes, located at the corners of the grid, were 3 mm in diameter. All other electrodes were 2 mm in diameter and spaced 4 mm apart. The platinum lead wires were tunneled to the subclavicular space and covered by a sterile dressing that allowed for connection to the neural recording system during recording sessions.

### Data collection

**Figure 2:**
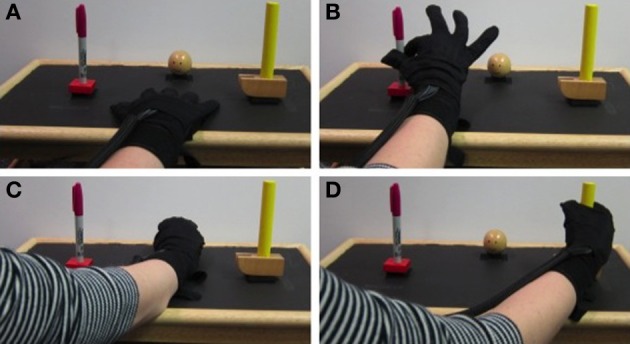
**Experiment setup**. Kinematics were recorded with a 5DT Data Glove while the subject (AE trials) or experimenter (AO trials) grasped 3 objects placed on a tray. The rest position is shown in **(A)**. Pinch, power, and cylindrical grasps are shown in **(B–D)** respectively.

ECoG signals were sampled at 1200 Hz and band-pass filtered between 0.1 and 200 Hz using a g.USBamp recording system (g.tec Guger Technologies OEG, Austria). BCI2000, a general-purpose software for BCI research, was used to record neural and kinematic data (Schalk et al., 2004). Able-bodied subjects were asked to execute and observe grasping movements while hand and finger kinematics were recorded, either from the participant or the experimenter, with a 14-sensor 5DT data glove (5DT Inc., Irvine, CA, USA). Throughout the manuscript, the two conditions are referred to as action execution (AE) and AO. Subjects performed the grasping task with the hand contralateral to the ECoG grids, which in all cases was the dominant hand. Three objects (ball, toy hammer, and pen) were fixed with Velcro to a tray in front of the subject in an arrangement that minimized arm movement. Each object required a different type of grasp: power grasp, cylinder grasp, and pinch, respectively. The objects and grasp postures are shown in Figure 2. Participants began each repetition with their hand resting on the tray approximately 8 inches away from the objects and were prompted by a computer screen which object to grasp. The computer screen was placed approximately 12 inches in front of the tray on a bedside table. The height was adjusted for each subject such that they could comfortably view the objects and screen simultaneously. An image of the object to be grasped was shown for 2.5 s with an interstimulus interval ranging from 2 to 2.5 s between repetitions. Subjects were instructed to begin the movement as soon as the image appeared. They were to grasp and hold the object without lifting it until the picture disappeared signaling them to return to the rest position. The return to rest was completed during the interstimulus interval.

The placement order of the objects was shuffled by the experimenter for each block of repetitions which consisted of 5 grasps per object. Three of the participants performed 15–20 trials for each object, while Subject I had 5 AE trials and 10 AO trials per object. The number of sessions, and thus trials, was determined by the duration of the subject's stay in the epilepsy monitoring unit. During the observation trials, the experimenter stood next to the subject and grasped the objects when prompted by the computer using the same hand as the subject used to perform the task. Subjects were instructed to simply watch the experimenter performing the task and the experimenter watched the subjects to ensure that their hand remained at rest. Subject S completed 10 AO trials per object on day 24 post-implant as described above. The experimenter grasped the objects with her right hand since Subject S had the ECoG array implanted over the left hemisphere.

### Data analysis and statistics

All analyses were performed using MATLAB (The Mathworks, Inc., Natick, MA, USA). Data were segmented into individual repetitions aligned to stimulus onset defined as the time at which the picture of an object appeared on the screen. Grasp onset was calculated by manual inspection of proximal interphalengeal (PIP) joint angles. Generally all of the fingers began to move together, but the earliest deviation from the rest angle for any finger was marked as grasp onset. Grasp completion time was defined when all PIP joints reached a constant angle signifying the steady-state grasp hold period. The spectral power of the neural data was computed from 0 to 200 Hz using the maximum entropy method with 3 Hz frequency bins (McFarland et al., 1997). The binned power spectra during each repetition were normalized by the average power recorded during all trials for a given electrode. This paper focuses on two frequency ranges: low frequency (10–40 Hz) and high frequency (65–115 Hz). The low frequency range corresponds to the mu and beta sensorimotor rhythms and the high frequency band represents the high-gamma band.

Data from all three grasps types were combined to form a single data set for each subject. For ease of comparison across participants, the trials were divided into three stages relative to stimulus onset: planning (0–0.5 s), early movement (0.5–1 s), and late movement (1–1.5 s). For each repetition, the normalized spectral power was calculated as the percent change relative to the average power recorded by a given electrode. For further analysis, the average normalized spectral power was calculated for the low and high frequency bands during the early movement phase. Depth of modulation is defined here as the average normalized spectral power in a given frequency band during the early movement phase. One-sided *t*-tests were used to test whether the mean depth of modulation measured during the early movement period was significantly different from 0. Essentially, this identified electrodes with significant activation during the early movement period as compared to the average activity recorded by a given electrode. Separate tests were completed for each electrode for both the low and high frequency band. Electrodes exhibiting a significant decrease in power in the low frequency band and a significant increase in power in the high frequency band were considered to have significant motor-related modulation.

The time series of normalized spectral power in the low and high frequency bands was calculated by averaging the normalized spectral power for all frequencies within the 10–40 and 65–115 Hz bands respectively (for an example, see Figure 4). For each subject, the normalized spectral power response for the low and high frequency band was averaged across trials for each object. The pairwise linear correlation coefficient was computed between the average time series of the normalized spectral power modulation during AE and AO for electrodes that showed a significant motor response during both AE and AO. The correlation coefficients for the modulated electrodes were combined across subjects into a single dataset so that the mean values could be computed for each object and frequency band. A fixed time window of 2 s following stimulus onset was used for this analysis. Two paired *t*-tests were used to test whether the depth of modulation of motor-related electrodes during the early movement period was significantly different between AE and AO for the low and high frequency bands.

## Results

### Kinematic data

Grasp onset for the 4 able-bodied subjects ranged from 423 to 665 ms after stimulus onset and grasp completion time ranged from 1312 to 1808 ms after stimulus onset. The mean onset time was 531 ± 65 ms for AE and 524 ± 59 ms for AO. Grasps were performed by the experimenter during the AO condition. The mean grasp completion times were 1594 ± 180 and 1502 ± 103 ms for AE and AO respectively. Grasp kinematics were generally similar for the 3 objects. For all grasps, participants extended their fingers slightly to open the hand prior to flexing the metacarpophalengeal (MCP) and PIP joints to achieve the final grasp position. For the pinch grasp, the middle, ring, and pinky fingers remain extended. The objects and grasp postures are shown in Figure 2.

### Identification of motor-related responses in able-bodied subjects

Electrodes that recorded a significant decrease in low frequency band (10–40 Hz) power and an increase in high frequency band power (65–115 Hz) during the early movement phase were considered to be motor-related electrodes. Of the 14 high-density ECoG electrodes for Subject F, 8 exhibited significant modulation during AE and 7 electrodes showed significant modulation during AO. Based on the projection of the x-ray onto a standard brain model (Figure 1), these electrodes were located over the middle frontal gyrus which contains the premotor cortex. For Subject G, 7 motor-related channels were identified during AE and 2 of these showed a significant modulation during AO. On the standard brain model, these electrodes spanned the pre- and post-central gyri which include the primary motor and somatosensory cortex respectively. Only 2 electrodes for Subject I (standard ECoG grid) showed a significant motor-related response during AE and none exhibited a significant response to AO. These electrodes appeared to be located over the pre-central gyrus. All 14 high-density ECoG electrodes were significantly modulated during AE and AO for Subject J. These electrodes were over premotor cortex spanning the superior and middle frontal gyrus. Figure 1 shows the location of significantly modulated electrodes for all able-bodied subjects. These electrodes covered various sensorimotor cortical areas across subjects, but all showed significant modulation during the motor task which involved observed or overt grasping.

### Comparison between action execution and observation

Table 1 summarizes the average depth of modulation for significantly modulated electrodes for each subject. For the able-bodied subjects who showed significant motor-related cortical activity during both AO and AE, the average depth of modulation of the low frequency band was 31.6 ± 10.6% in the AO condition and 42.7 ± 21.6% in the AE condition. For the high-gamma band, the average depth of modulation was 19.9 ± 7.7% in the AO condition and 25.0 ± 11.3% in the AE condition. The depth of modulation was significantly greater during AE for both the low (*p* = 0.002) and high frequency band (*p* = 0.02).

**Table 1:** **Average depth of modulation for electrodes significantly modulated by AE and AO**.

**Subject**	**Low frequency band**		**High frequency band**	
	**AE**	**AO**	**AE**	**AO**
F	14.6 ± 4.6%	25.0 ± 5.3%	28.2 ± 17.3%	18.7 ± 8.6%
G	25.9 ± 13.7%	15.8 ± 5.9%	34.7 ± 10.1%	12.2 ± 3.7%
I	20.3 ± 0.1%	–	24.6 ± 12.4%	–
J	57.2 ± 9.8%	36.8 ± 7.7%	22.2 ± 7.4%	21.4 ± 7.3%
S	–	13.6 ± 2.7%	–	22.6 ± 11.4%

Subject S was unable to complete the AE condition due to his spinal cord injury and Subject I did not exhibit significant modulation during AO.

**Figure 3:**
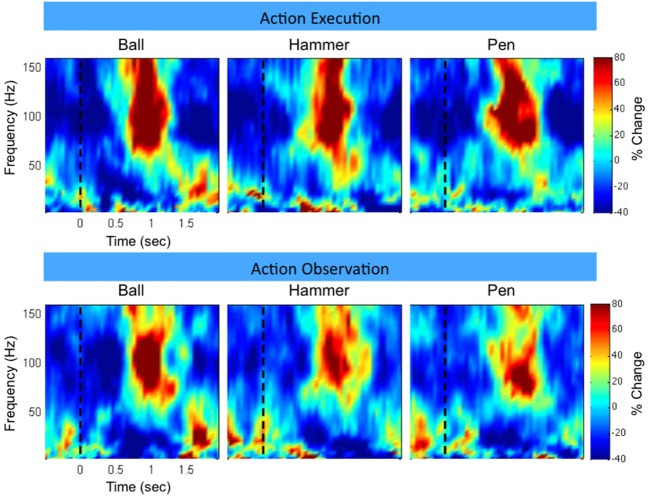
**Example neural activity during action execution (AE) and observation (AO)**. Example time-frequency plot of neural activity (Subject F, high-density ECoG grid electrode #7) during AE and AO of three grasping tasks (Ball, Hammer, Pen). The color indicates normalized spectral power expressed as the percent change from the average activity of a given electrode. For both conditions, a decrease in low frequency spectral power and an increase in high frequency spectral power were observed with a similar temporal profile. Data was averaged based on stimulus onset (shown as a vertical dotted line at *t* = 0).

**Figure 4:**
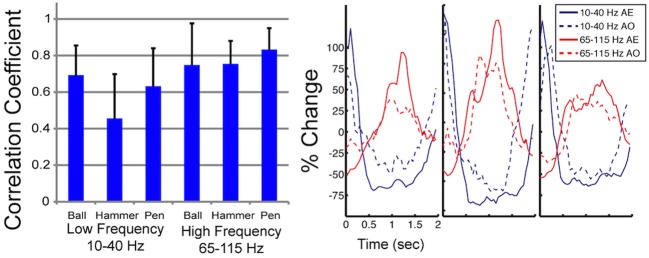
**Correlation in spectral power during AE and AO**. On the left, the mean correlation between normalized spectral power of the low and high frequency bands measured during AE and AO for three grasp conditions is shown. Correlations were computed for each of the 22 electrodes, from 3 subjects, that exhibited a significant motor response during both AE and AO and then the correlation coefficient values were averaged. Error bars represent the standard deviation. On the right, a sample plot of the average modulation of the low and high frequency bands is shown (Subject J, high-density ECoG electrode #8). Time 0 indicates stimulus onset. Normalized spectral power is reported as the percent change in power from the average response recorded by this electrode over all repetitions.

Figure 3 shows time-frequency plots of the high-density ECoG activity recorded by an electrode for Subject F during AE and AO. During both conditions, for all grasps, the low frequency band power decreases and the high frequency band power increases (relative to each band's average power throughout an experiment session) immediately prior to and during movement. To quantify this similarity, the average low and high frequency power was computed over the trial duration and then averaged across trials for each object. Figure 4 shows the time course of the normalized power in the low and high frequency bands for a sample electrode that was significantly modulated during AE and AO. This figure illustrates the similarity of the modulation of spectral power during both conditions. For this particular subject, an increase in low frequency band power was recorded near the time of stimulus onset. Analysis of the kinematic data revealed that this increase corresponded to the low frequency band power rebound (Jurkiewicz et al., 2006) following movement back to the center resting position which occurred during the interstimulus interval. Figure 4 also illustrates the average correlation in normalized spectral power measured from all significantly modulated electrodes during AE and AO. Pairwise correlations of the average low and high frequency band power time series recorded during AE and AO for each object were computed for all channels that showed a significant motor-related response for a fixed time window of 2 s after stimulus onset. In general, the correlation values indicate a strong similarity in spectral power modulation between conditions. Across all objects, correlations were stronger for the high-gamma band (*r* = 0.779 ± 0.17) than for the low frequency band (*r* = 0.598 ± 0.22). For comparison, unmodulated electrodes on the high density ECoG grid or standard ECoG grids (*n* = 220) showed average correlations of 0.295 ± 0.20 for the high frequency band and 0.242 ± 0.18 for the low frequency band.

**Figure 5:**
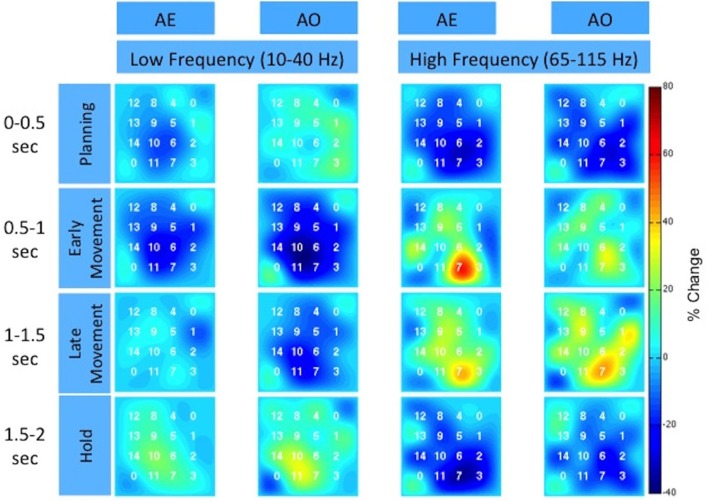
**Spatiotemporal distribution of the low and high frequency band across the high-density ECoG grid for Subject F**. The time intervals are relative to stimulus onset. The color indicates the percent change in the spectral power normalized to the average activity recorded by the electrode over all repetitions. The two corner electrodes marked as “0” served as reference channels.

To further illustrate the similarity between AE and AO, Figure 5 and the Supplemental Movie 1 show the temporal evolution of the spatial distribution of the low and high frequency band responses across the high-density ECoG grid. Data was averaged across the three grasp types. Many channels on the high-density ECoG grid show a low frequency band power decrease followed by the typical rebound, which is an increase in low frequency band power after the movement ends (Jurkiewicz et al., 2006). The greatest power decrease of the low frequency band appeared to be centered at electrode 10 for both AE and AO. During the early and late movement periods, the high frequency band exhibits an increase in power for both AE and AO. Electrode 7 recorded the largest increase in high frequency band power for both conditions.

### Motor-related response to action observation after spinal cord injury

**Figure 6:**
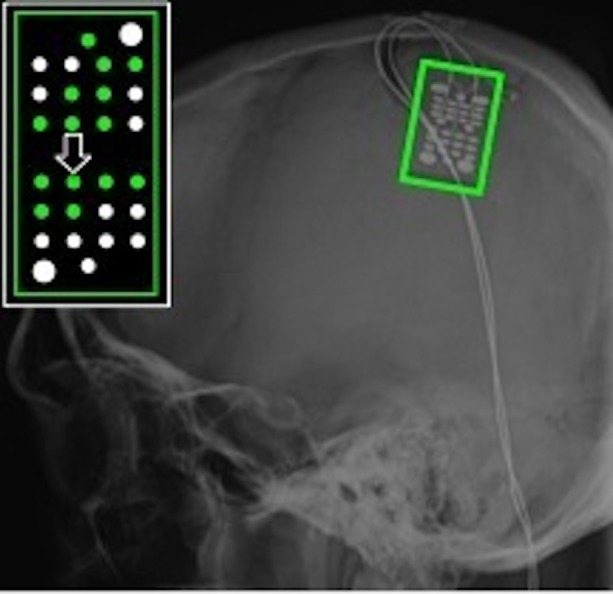
**ECoG grid location for Subject S, an individual with C4 complete spinal cord injury**. The schematic inset shows electrodes that recorded a significant motor-related response during AO highlighted in green. The majority of these electrodes were located over primary somatosensory cortex. The large arrow marks the electrode whose data are shown in Figure 7.

**Figure 7:**
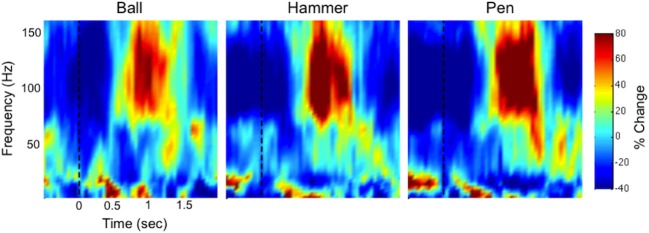
**Example time-frequency plot of neural activity (Subject S, electrode #12 as marked in Figure 6) during action observation (AO) of three grasping tasks (Ball, Hammer, Pen)**. The color indicates normalized spectral power expressed as the percent change from the average activity of a given electrode. In a person with spinal cord injury, we observed a decrease in low frequency spectral power and an increase in high frequency spectral power that was similar to the responses observed in able-bodied subjects (Figure 3).

The experimenter performed all grasps during AO trials with a mean grasp onset time of 561 ± 76 ms and a mean grasp completion time of 1674 ± 170 ms after stimulus onset. Fourteen of the 28 recording electrodes showed a significant motor-related response during AO. Electrodes showing significant motor-related modulation are shown on x-ray in Figure 6. When co-registered to the subject's structural MRI (shown in Wang et al., 2013), it appears that the majority of electrodes are located over primary somatosensory cortex. This reflects somatosensory activation resulting from observed grasping. The modulated electrodes were localized to contiguous electrodes on the medial and central portion of the ECoG grid. The average depth of modulation during AO was −13.6 ± 2.7% for the low frequency band and 22.6 ± 11.4% for the high-gamma band. Figure 7 shows time-frequency plots of the cortical activity recorded by an electrode for Subject S during AO of grasp. Cortical activation recorded with ECoG during AO was similar to that observed for able-bodied subjects.

## Discussion

This paper provides evidence that observation-related activity can be recorded from the cortical surface with ECoG. To our knowledge this is the first study to document congruent activity in the high-gamma band during overt and observed movement. The high-gamma band carries significant information about movement, both overt and covert, and therefore may be important for developing ECoG-based BCI systems (Leuthardt et al., 2004; Miller et al., 2010; Wang et al., 2010b). This work extends the findings of others who observed congruence in the low frequency band during AE and AO in MEG and EEG studies (Hari et al., 1998; Muthukumaraswamy et al., 2004; Caetano et al., 2007; Perry and Bentin, 2009; Press et al., 2011). We observed that the temporal evolution of activity was similar during both conditions in the low and high frequency bands as evidenced by the strong correlations in normalized spectral power over the planning and movement phases (Figure 4).

Many investigators believe that observation-induced cortical activity is facilitated by the mirror neuron system which is a group of neurons that are activated similarly when a person acts and when the person observes the same action performed by another person (Fabbri-Destro and Rizzolatti, 2008). The mirror neuron system formally includes a subset of neurons in the inferior parietal lobe, ventral premotor cortex, and inferior frontal gyrus. Early studies of the mirror neuron system show that these neurons are selective for different grasp types (Gallese et al., 1996) and are maximally engaged for movements involving interaction between the hand and an object. Previous studies have also used kinesthetic motor imagery, during which a person imagines themselves performing an action, to activate the motor cortex in the absence of overt movement (McFarland et al., 2000; Ehrsson et al., 2003; Miller et al., 2010). We were able to estimate the location of modulated electrodes using the LOC toolbox (Miller et al., 2007). Although there are limitations with estimating electrode position from a single planar x-ray after a neurosurgical intervention, these results show that the modulated electrodes were over pre-motor, primary motor, or primary somatosensory cortex. Premotor cortex is formally part of the mirror neuron system and so one would expect that AO would result in modulation of these electrodes. Other studies have also shown that primary motor and somatosensory cortex are modulated during AO, although there is no clear consensus as to the mechanism of this activation (Hari et al., 1998; Avikainen, 2002; Caetano et al., 2007). Action observation and motor imagery likely leads to an internal representation of movement, which may activate the mirror neuron system (Jeannerod, 2001). It is possible that the mirror neuron system facilitates the imagination- or observation-related sensorimotor activity observed in this and other studies.

Previous studies have found that the depth of modulation for the low frequency band is lower during AO compared to AE (Hari et al., 1998; Caetano et al., 2007) and our results were generally consistent with this finding as summarized in Table 1. Some channels did show a larger modulation during the early movement period of AO compared to AE, however the magnitude of the difference was small. Overall, the depth of modulation for both the low frequency band and high-gamma band was significantly greater during AE compared to AO. For the high-gamma band, the average depth of modulation in able-bodied subjects was 19.9 ± 7.7% in the AO condition and 25.0 ± 11.3% in the AE condition, approximately 5% greater. Two of the participants (G and J) showed greater modulation of the low frequency band during AE. The average increase in depth of modulation was 10.2 ± 7.8% for Subject G and 20.4 ± 5.4% for Subject J. Interestingly, Subject F showed greater modulation of the low frequency band during AO compared to the AE condition, while high frequency band modulation was 9.5 ± 11.8% lower during AO. For this subject, we observed a similar spatiotemporal evolution of the low and high frequency neural activity during these two conditions (Figure 5). Visual inspection of the data on a single channel basis (Figures 3, 4) highlights the similarity in the cortical activation pattern between AE and AO as well as between grasp types.

For the purposes of developing a motor-BCI training strategy, we are interested studying methods to access motor-related activity in the absence of overt movement. To date, most ECoG studies have been conducted in patients undergoing intracranial monitoring for intractable epilepsy. We were able to collect ECoG data during AO from an individual with SCI when he participated in a BCI study with our research group (Wang et al., 2013). Approximately half of the electrodes on the ECoG grid implanted over his sensorimotor cortex were modulated by observed hand grasps. This is the first time that significant action observation-related activity has been measured with ECoG in someone with chronic tetraplegia. This response was observed on contiguous electrodes on the ECoG grid suggesting that the somatotopic representation of movement and the related somatosensory response may be preserved after SCI. Additionally, the spatiotemporal pattern and depth of modulation was similar for the motor-related responses recorded from the spinal cord injured subject and the able-bodied participants. These results are in line with previous studies that have shown that motor-related activation during covert movement is a robust and repeatable response among able-bodied individuals (Hari et al., 1998; Ehrsson et al., 2003; Neuper et al., 2005; Tkach et al., 2007; Miller et al., 2010). Previous studies with fMRI have shown that observation-related motor activity is still present after SCI (Sabbah et al., 2002). Further, recent evidence suggests that observation or imagery-related paradigms for BCI decoder training may be effective even after long-term paralysis (Hochberg et al., 2006; Kim et al., 2008; Collinger et al., 2013b). In these studies, subjects are often asked to imagine performing a movement while observing the visual display because motor imagery is known to activate motor cortex in a similar manner as overt movement. The ability to measure grasp-related activity through observed movement would facilitate BCI decoder training for controlling assistive devices for restoring or augmenting hand grasp such as a functional electrical stimulator or prosthetic hand. Restoration of upper limb function has repeatedly been identified as a top priority for individuals with tetraplegia (Anderson, 2004; Collinger et al., 2013a). Previous studies decoded detailed hand kinematics using ECoG signals recorded during overt hand movement (Kubánek et al., 2009; Wang et al., 2009) and an important next step will be to decode observed hand movement using ECoG data collected during AO. Further, it may be interesting to quantify differences in neural activation patterns between AO and AE whereas in this study the goal was to identify similarities between these two conditions.

The current study has several limitations. First, it studied a small number of subjects, including four able-bodied subjects who were undergoing ECoG monitoring for intractable epilepsy and one subject with tetraplegia. The number of subjects is comparable to previous ECoG studies in individuals undergoing ECoG monitoring (Schalk et al., 2007; Ball et al., 2009; Kubánek et al., 2009), and AO-related high-gamma band activity was captured in both able-bodied individuals and a person with chronic paralysis. Second, ECoG data reported here were collected either from a standard clinical grid or a high-density research grid. ECoG grid placement in subjects undergoing epilepsy monitoring was dictated by clinical needs, and the high-density research grid was placed over cortical areas not covered by clinical grids. The high-density research grids offer better spatial resolution than standard clinical grids, and are potentially more sensitive to subtle changes in local cortical activity (Wang et al., 2009; Slutzky et al., 2010; Wodlinger et al., 2011). In the current study, both high-density grids captured AO-related high-gamma band modulation. Last, one of the participants (Subject I) did not exhibit significant observation-related activity, even though two electrodes recorded a significant motor-related response during AE. A decrease in low frequency band power was observed in these channels during AO, although this was not statistically significant during the early movement phase. As shown in Figure 1, the position of the electrodes for Subject I is more medial than for the other subjects It is possible that the small number of repetitions (*n* = 10) may have limited our ability to measure a significant motor response during AO for this subject. This subject was much younger than the other subjects may have been less engaged during the AO task.

It is possible that eye movement can result in ECoG signal modulation, but this was mitigated in several ways. Eye movement often produces a transient time-domain response in scalp EEG recordings which results in an increase in power in the very low frequencies while high-gamma band activity resulting from eye movement is spatially restricted to the occipital lobe (Nagasawa et al., 2011; Plochl et al., 2012). We limited our analysis to the early movement phase when we expect that the subject's eyes were fixed on the object being grasped. The experimental setup was designed so that subjects could view the computer screen and objects with minimal eye movement and the object positions were varied between experimental blocks. Further, we would not expect that eye movement artifacts would be limited to localized cortical areas. We found that AE and AO resulted in modulation of focal areas of cortex in the pre-motor, motor, and somatosensory cortex. Finally, for the high-density ECoG grids, we use a corner electrode as a common reference which minimizes artifacts from sources away from the grid location.

ECoG recording in able-bodied subjects revealed congruent high-gamma band activity and sensorimotor rhythm modulation between observed and overt hand movements. Additionally, robust modulation of the high-gamma band and sensorimotor rhythm during AO was recorded from a participant with tetraplegia. These results suggest that AO can serve as a useful paradigm for neural decoder training for ECoG-based BCI applications to restore function to individuals with chronic paralysis.

## Author contributions

Jennifer L. Collinger, Douglas J. Weber, Michael L. Boninger, Elizabeth C. Tyler-Kabara, and Wei Wang designed the study. Jennifer L. Collinger, Ramana Vinjamuri, Alan D. Degenhart, Gustavo P. Sudre, and Wei Wang collected the data. Jennifer L. Collinger, Ramana Vinjamuri, Alan D. Degenhart, and Gustavo P. Sudre contributed to data analysis. Ramana Vinjamuri, Alan D. Degenhart, Gustavo P. Sudre, and Wei Wang developed the software used for data collection. Jennifer L. Collinger and Wei Wang wrote the manuscript and all authors provided critical review and approval of the manuscript.

### Conflict of interest statement

The Associate Editor Martin Oudega declares that, despite being affiliated to the same institution as author(s) Jennifer L. Collinger, Ramana Vinjamuri, Douglas J. Weber, Michael L. Boninger, and Wei Wang, the review process was handled objectively and no conflict of interest exists. The authors declare that the research was conducted in the absence of any commercial or financial relationships that could be construed as a potential conflict of interest.

## Abbreviations

AO: action observation
AE: action execution
ALS: amyotrophic lateral sclerosis
BCI: brain-computer interface
EEG: electroencephalography
ECoG: electrocorticography
fMRI: functional magnetic resonance imaging
MCP: metacarpophalengeal
MEG: magnetoencephalography
PIP: proximal interphalengeal.
